# Attention Deficits and ADHD Symptoms in Adults with Fabry Disease—A Pilot Investigation

**DOI:** 10.3390/jcm10153367

**Published:** 2021-07-29

**Authors:** Nadia Ali, Amanda Caceres, Eric W. Hall, Dawn Laney

**Affiliations:** 1 Department of Human Genetics, Emory University School of Medicine, Atlanta, GA 30322, USA; Dawn.Laney@emory.edu; 2 AdventHealth Cancer Institute, Orlando, FL 32804, USA; Aehodgkins24@gmail.com; 3 Department of Epidemiology, Rollins School of Public Health, Emory University, Atlanta, GA 30322, USA; Eric.W.Hall@emory.edu

**Keywords:** Fabry disease, attention, Attention Deficit/Hyperactivity, cognition

## Abstract

The present pilot study examines subjective reported symptoms of attention-deficit/hyperactivity (AD/H) in adults with Fabry disease (FD) in comparison with existing normative control data. Existing data from 69 adults with FD via the Achenbach System of Empirically Based Assessment Adult Self-Report questionnaire were analyzed. The results demonstrated a higher prevalence of AD/H symptoms in adults with FD than in the general United States population, with a roughly equal endorsement of Inattention/Attention Deficit symptoms (AD), Hyperactivity-Impulsivity (H-I) symptoms, and Combined Inattention/hyperactivity-impulsivity (C) symptoms. No gender differences were observed. While all subjects endorsing H-I symptoms fell into the symptomatic range on the AD/H scale, only two-thirds of subjects endorsing AD did so. This suggests that attention difficulties with FD are not solely explained by ADHD. Adults with FD who endorsed the AD, H-I, and C symptoms were also more likely to report mean adaptive functioning difficulties. These findings support the growing literature regarding attention difficulties in adults with FD, as well as suggesting a previously unrecognized risk of AD/H symptoms. Future research involving the objective assessment of ADHD in adults with FD is recommended. When serving adults with FD clinically, healthcare professionals should address multiple areas of care, including physical, psychological, and cognitive arenas.

## 1. Introduction

Fabry disease (FD) is an X-linked lysosomal storage disorder (LSD) caused by mutations in the *GLA* gene, leading to a deficiency of α-galactosidase A (α-gal A; EC 3.2.1.22) and resulting in the storage of globotriaosylceramide (GL3) and related lipids in the lysosome. Its incidence has historically been estimated at 1:40,000 male live births; however recent data suggests as high as 1:3000 [1], with a range of 1250–117,000 worldwide [2]. The symptoms and complications include acroparesthesia, fatigue, anhidrosis, angiokeratomas, gastrointestinal symptoms, kidney failure, cardiovascular problems, and stroke [3,4,5,6,7]. The standard of care treatment is enzyme replacement therapy (ERT) or chaperone therapy (in individuals with amenable *GLA* mutations).

Historically, research has focused on somatic manifestations of FD, with less attention paid to neuropsychological manifestations. However, recent research suggests difficulties with cognitive functioning, particularly in the realm of attention and concentration, with implications for central nervous system (CNS) functioning in patients with FD.

The initial neuropsychological screening studies of patients with FD reported contradictory results due to varying testing methods and small sample sizes. One initial study found patients with FD performed marginally better on tasks of attention than normal controls and slightly worse on tasks measuring language skills, with unimpaired performances in other cognitive domains [8], while another found patients with FD performed mildly worse on tasks of attention than normal controls [9]. Although the patients initially appeared to perform worse on executive functioning tasks, this difference disappeared once corrected for the effects of depression and remained absent in a subset of patients eight years later [10]. The subsequent early research found that patients with FD performed worse on some tests of attention (especially those involving information processing speed) [11,12,13], as well as some measures of executive functioning [11,13]. 

The first study to examine neurocognitive functioning in FD using comprehensive and well-validated neuropsychological measures rather than screening tools found that males with FD demonstrated a slower information processing speed and reduced performance on measures of executive functioning compared to both females with FD and 15 age-matched normal controls [14]. However, several confounds were present. None of the females with FD had experienced a stroke or transient ischemic attacks compared to 33% of the males. Males with FD were also more likely to report symptoms of anxiety and depression, which is known to have delirious effects on cognition, including attention, memory, and executive functioning [15]. Taken together with a low sample size and correlational analyses suggesting a link between the cognition and clinical measures of disease severity, these confounds compromised the generalizability. 

A more recent study found 29.3% of Danish patients with FD to have cognitive difficulties, with attention, psychomotor speed, and executive functioning once again being the most frequently impaired [16]. Neither depression, disease severity, nor gender predicted objective cognitive impairment; however, depression was associated with the subjective perception of cognition. The subjective perception of cognition was lower than the actual cognitive performance among subjects. 

In comparison, subjective perceptions of cognitive impairment among Dutch subjects with FD were found to be much greater (64%) than the objective evidence of impairment (16%) [17]. Objective impairment was found primarily in males, especially those with classical FD. Follow-up testing one year later, however, demonstrated a worsening objective cognitive impairment in only 5.3% of subjects and was found more often among women (three women and one man) [18]. Subjective impairment was prevalent in both genders and correlated with depression [16,17]. 

Given the increasing evidence of the role of FD in aspects of attention, anecdotal patient reports regarding the use of medication for Attention Deficit Hyperactivity Disorder (ADHD) should perhaps not come as a surprise. Previously referred to as Attention Deficit Disorder (ADD) in the Diagnostic and Statistical Manual of Mental Disorders 3rd Edition (DSM III) [19], one of the core symptoms is a deficiency in attention. The updated label of ADHD in DSM IV and DSM-5 is an umbrella term for a wide range of symptoms and consists of three main types: Inattentive/Attention Deficit (AD), Hyperactive-Impulsive (H-I), and Combination (C) types [20,21]. While attention-deficit/hyperactivity (AD/H) symptoms in patients with FD have been shown to be associated with poorer adaptive functioning (AF) [22], no further exploration of AD/H symptoms in FD has been done. A pilot study specifically documenting and exploring patient reports of attention deficits will be beneficial as a prequel to more in-depth studies of attention deficits in patients with FD.

The present pilot study examines the self-reported symptoms of attention deficits/hyperactivity in adults with FD in comparison with the existing normative control data, as well as potential differences in the frequency between symptoms of attention deficits and symptoms of hyperactivity. In addition, we explored the possible association between attention-deficit/hyperactivity symptoms and poorer adaptive functioning in patients with FD. 

## 2. Materials and Methods

Data was derived from a subset of data in existence at the Emory Lysosomal Storage Disease Center. Specifically, data concerning Attention Deficit/Hyperactivity, Attention, Inattention, Hyperactivity-impulsivity, Somatic Symptoms, Depression, Anxiety, and Mean Adaptive Functioning were utilized from the Achenbach System of Empirically Based Assessment (ASEBA) Adult Self-Report (ASR) questionnaires completed by patients with FD between January 2005 and July 2013. Approval from the Institutional Review Board was granted through Emory University (IRB00068700).

The ASEBA ASR is a reliable, validated measure of social-adaptive and psychological functioning in adults aged 18–59 and the OASR for ages 60–90+ [23]. Norms represent the mix of ethnicities, socioeconomic status, urban–rural–suburban residency, and geography within the US. Raw scores are converted to T-scores to permit comparisons with the general population. Scale scores are normed by gender and age and categorized as normal (<93rd percentile), borderline-clinical (93rd–97th percentiles), or clinical (>97th percentile). The ASEBA is used with a wide variety of medical conditions, including cystic fibrosis, Fabry, Morquio, Turner, Williams, Angelman, and Prader-Willi syndromes [22,23,24,25]. 

### Data Analysis

ASEBA ASR raw data was entered into assessment data manager (ADM, version 9.0) ASEBA scoring software (https://adm-assessment-data-manager.software.informer.com/9.0/, accessed on 1 June 2021), which produces detailed profiles on multiple aspects of psychological functioning. For this study, data from the DSM-Oriented Scale for AD/H, as well as the Attention Problem Syndrome scale, Depression scale, Somatic Complaints scale, and Mean Adaptive Functioning scale, were utilized. Subjects with T-scores in the borderline-clinical and clinical ranges were considered to have symptoms for the purposes of this study. 

All data analysis was done using SAS 9.4 (SAS Institute, Cary, NC, USA). Demographic participant characteristics were summarized using frequencies and proportions. Chi-square and Fisher’s exact tests were used to assess the associations between mean adaptive functioning and demographic variables of interest. Similarly, chi-square tests were used to assess the association between depressive symptoms and gender, AD/H symptoms, H-I symptoms, and AD symptoms. The prevalence of AD/H in our study sample was compared to the most recent estimated prevalence of AD/H among the US adult population [26] using Fisher’s exact test. All statistical tests were assessed using an alpha = 0.05.

## 3. Results

Existing data from 69 adults with FD who completed the ASEBA ASR questionnaire was examined. The demographic information is presented in Table 1. The ages ranged from 18 to 61 years.

**Figure 1:**
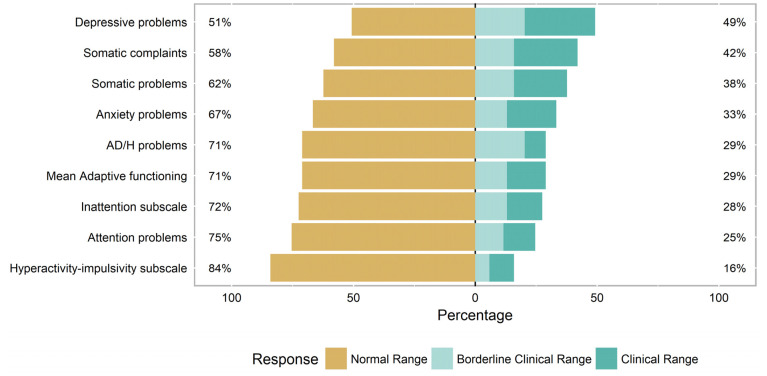
Prevalence of ASEBA symptoms in adults with Fabry disease.

Of the 69 subjects who completed the ASEBA ASR, twenty (29%) endorsed symptoms within the borderline-clinical-to-clinical range on the AD/H problems scale (Figure 1). This represents a significantly higher prevalence of AD/H symptoms in our population of adults with FD than in the general population (*p* < 0.001), using the most recently estimated prevalence (4.4%) of adult ADHD in the United States [26].

Among the twenty subjects endorsing symptoms within the borderline-clinical-to-clinical range on the AD/H scale, the source of those scores was almost equally balanced between the symptomatic endorsement of AD items, H-I items, and combined AD/H items, with a final three subjects whose endorsement of items was evenly split such that they fell within the normal ranges on the individual subscales while still falling within the symptomatic range on the overall combined AD/H scale (Table 2). All subjects endorsing the H-I symptoms within borderline-clinical-to-clinical range also scored in the borderline-clinical-to-clinical range on the AD/H scale; however, only 12/19 (63.2%) subjects endorsing AD symptoms also scored in the borderline-clinical-to-clinical range on the AD/H scale.

Almost half of the adults with FD (49%) were also noted to self-report depressive symptoms in the borderline-clinical-to-clinical range on the ASEBA ASR, with no significant differences between the male and female subjects (*p* = 0.537). A third of the adults with FD (33%) self-reported symptoms of anxiety, with no significant differences between the male and female subjects (*p* = 0.0870). Almost a third of adults with FD (29%) self-reported difficulties in adaptive functioning, with no significant differences between the male and female subjects (*p* = 0.060). Over a third of adults with FD (38%) self-reported somatic symptoms in the borderline-clinical-to-clinical range, with no significant differences between the male and female subjects (*p* = 0.2468).

Adults who scored in the borderline-clinical-to-clinical range on the AD/H scale, AD subscale, and H-I subscale were significantly more likely to self-report both depressive symptoms and somatic problems (Table 3). Adults scoring in the borderline-clinical-to-clinical range on the AD/H scale and H-I scale were significantly more likely to self-report anxiety symptoms as well (Table 3). There were no differences between males and females in any of these categories.

There were no significant demographic differences between those with and without AF deficits; however, the adults with FD who self-reported AD problems, AD/H symptoms, depressive symptoms, and anxiety were also significantly more likely to report AF difficulties (Table 4).

## 4. Discussion

The present study is a pilot exploration of self-reported attention deficit symptoms in adults with Fabry disease. The results demonstrate a higher prevalence of AD/H symptoms in adults with FD than in the general United States population, with a roughly equal numbers of adults with FD endorsing AD symptoms, H-I symptoms, and Combined symptoms. While ADHD is more common in men than women in the general population [26], and some studies have found greater evidence of cognitive impairment in men with FD than women [14,17], our study found no gender differences in the rate of AD/H, H-I, or AD symptoms amongst adults with FD.

While all subjects endorsing H-I symptoms fell within the symptomatic range on the AD/H scale, only two-thirds of subjects endorsing AD symptoms did so. The remaining third endorsing AD without AD/H symptoms suggests that attention difficulties within FD are not solely linked to AD/H and lends credence to prior research outlining cognitive difficulties in attention in FD [9,11,13,16]. However, the reverse is also true; the endorsement of an equally high rate of H-I symptoms among our FD population suggests a previously unrecognized prevalence of such symptoms among adults with FD separate from attention deficits.

Of note, almost half of adults with FD in the present study endorsed symptoms of depression (49%), with no significant differences between men and women. This replicates the previously reported high rates of depression among adults with FD, with prevalence estimates ranging from 15% to 62% [9,13,25,27,28,29]. The present study likewise supported research demonstrating that depression in FD does not follow gender norms, with males reporting equal or greater rates than females [14,27]. While the most common factor associated with depression in FD is chronic pain [13,27,30], economic status, relationship status, specific coping styles, and somatic symptoms such as anhidrosis and acroparaesthesia have also been associated with depression and a lower QOL [27,30,31]. 

While depression can have deleterious effects on attention [15], its interaction with hyperactivity-impulsivity goes in the opposite direction; it is more likely to be a consequence of ADHD than a cause. Thus, while adults with FD who reported symptoms of AD/H, AD and H-I were more likely to also report symptoms of depression, this is consistent with previous research demonstrating that people with ADHD are at risk for depression and anxiety as a result of living with ADHD [26,32,33,34].

Finally, the present study found adults with FD-endorsing AD symptoms, AD/H symptoms, depressive symptoms, and anxiety were also significantly more likely to endorse adaptive functioning (AF) difficulties. An indication of the effectiveness with which individuals cope with the demands of everyday tasks and responsibilities as parents, students, employees, etc., AF is measured via evaluations such as the ASEBA focused on individuals’ relationships, jobs, education, substance use, psychological issues, and coping skills. These findings corroborate earlier research in which FD patients had a higher rate of mean AF deficits compared to population norms, with poorer AF associated with greater rates of AD/H, depression, and anxiety [22]. 

All of the above findings make clear the need to pay attention to the psychological symptoms associated with FD, including the possibility of symptoms of AD/H, and expand our standard of care to include mental health treatments, if necessary. Of note, of the 20 people who self-reported AD/H symptoms in our sample, four had been prescribed medication typically used for ADHD at some point in their life, though only one had undergone a clinical diagnosis for their symptoms. As symptoms of ADHD are more heterogeneous and subtle in adults than children [35,36], with only 25% of adults with ADHD receiving treatment [26], it is possible that ADHD symptoms in adults with FD are being overlooked amidst the urgency of the other symptoms of FD. 

The limitations of this study include the use of self-reported symptoms at a single point in time; however, adults with ADHD have been found to be quite reliable in identifying their own symptoms via self-reported measures [35], and an earlier study found that adults with FD were, if anything, more likely to underreport than overreport neurocognitive complaints [16]. Another limitation is the comparison between self-reported symptoms (FD population) and diagnosis (US population). To our knowledge, there is no nationally representative database of self-reported symptoms of AD/H, as compared to the frequency of diagnosis. Previous research has likewise used self-reported ADHD symptoms rather than diagnoses and presented evidence for the use of such as an effective tool [37]. Finally, this study included data primarily from Caucasian adults with FD and may not be generalizable to adults with FD of other ethnicities.

The implications of this study include the need for greater attention to cognitive and psychological health in people with FD, particularly in the areas of attention, AD/H-like symptoms, depression, anxiety, and adaptive functioning. Genetic counselors and other healthcare providers should address such issues in their annual clinic appointments and make referrals as needed to maximize overall treatment for patients with FD. 

The recommendations for future research include a more objective assessment of AD/H symptoms in patients with FD, as well as further in-depth neurocognitive studying of attention/concentration in FD. Such research should utilize objective neuropsychological tests with the existing normative data with the general population.

## 5. Conclusions 

In conclusion, the present study suggests that adults with FD are at a higher risk than the general population for attention deficits, as well as symptoms of ADHD, with equal rates among men and women. When serving adults with FD clinically, genetic counselors and other healthcare professionals should address multiple areas of care, including the physical, psychological, and cognitive issues that may accompany the disease.

## Figures and Tables

**Table 1:** Demographic characteristics of the subjects.

	*n*	%
Gender		
Female	38	55.1
Male	31	44.9
Race		
African American	4	5.8
Caucasian	61	88.4
Other	4	5.8
Education		
Some High School	5	7.3
High School or GED degree	13	18.8
Some college	24	34.8
College degree or higher	27	39.1
Employment		
Yes	32	46.4
No	23	33.3
Disability	4	5.8
Student	10	14.5

**Table 2:** Subscale breakdown among adults with FD endorsing AD/H symptoms (*n* = 20).

Subscale within Symptomatic Range	*n*	Column%
Combined Attention Deficit/Hyperactivity	6	30.0
Attention Deficit/Inattention only	6	30.0
Hyperactivity-impulsivity only	5	25.0
None (items evenly split)	3	15.0

**Table 3:** Association between the comorbid symptoms and symptoms of AD/H, AD, and H-I in adults with FD when using the ASEBA Adult Self-Report.

		AD/H					Inattention					Hyperactivity-Impulsivity				
		Clinical/Borderline		Normal			Clinical/Borderline		Normal			Clinical/Borderline		Normal		
	*N*	*n*	col %	*n*	col %	*p*-Value	*n*	col %	*n*	col %	*p*-Value	*n*	col %	*n*	col %	*p*-Value
Depressive Problems	34	17	85.0	17	34.70	<0.001	15	79	19	38.0	0.002	10	90.9	24	41.4	0.003
No depressive problems	35	3	15.0	32	65.30		4	21.1	31	62.0		1	9.1	34	58.6	
Anxiety problems	23	13	65.0	10	20.4	<0.001	9	47.4	14	28.0	0.127	7	63.6	16	27.6	0.034
No anxiety problems	46	7	35.0	39	79.6		10	52.6	36	72.0		4	36.4	42	72.4	
Somatic problems	26	12	60.0	14	28.6	0.015	11	57.9	15	30.0	0.033	8	72.7	18	31.0	0.015
No somatic problems	43	8	40.0	35	71.4		8	42.1	35	70.0		3	27.3	40	69.0	
Female	38	13	65.0	25	51	0.290	8	42.1	30	60.0	0.182	7	63.6	31	53.5	0.743
Male	31	7	35.0	24	49		11	57.9	20	40.0		4	36.4	27	46.6	

**Table 4:** Association between psychological symptoms and adaptive functioning in adults with FD when using the ASEBA Adult Self-Report.

		Mean Adaptive Functioning				
		Normal Range		Borderline/Clinical Range		
Demographic Characteristics	*N*	*n*	Row %	*n*	Row %	*p*-Value
Sex						0.06
Female	38	23	60.5	15	39.5	
Male	31	26	83.9	5	16.1	
Race						
African American	4	3	75	1	25	0.137
Caucasian	61	45	73.8	16	26.2	
Other	4	1	25	3	75	
Education						0.269
Some High School	5	3	60	2	40	
High School or GED degree	13	7	53.9	6	46.2	
Some college	24	17	70.8	7	29.2	
College degree or higher	27	22	81.5	5	18.5	
Employment						0.702
Yes	32	23	71.9	9	28.1	
No	23	15	65.2	8	34.8	
Disability	4	4	100	0	0	
Student	10	7	70	3	30	
ASR Conditions						
Attention problems	17	5	29.4	12	70.6	<0.001
Normal range	52	44	84.6	8	15.4	
AD/H problems	20	7	35	13	65	<0.001
Normal range	49	42	85.7	7	14.3	
Somatic problems	26	15	57.7	11	42.3	0.099
Normal range	43	34	79.1	9	20.9	
Depressive problems	34	15	44.1	19	55.9	<0.001
Normal range	35	34	97.1	1	2.9	
Anxiety problems	23	11	47.8	12	52.2	0.005
Normal range	46	38	82.6	8	17.4	
Somatic complaints	29	17	58.6	12	41.4	0.065
Normal range	40	32	80	8	20	
H-I subscale	11	3	27.3	8	72.7	0.001
Normal range	58	46	79.3	12	20.7	
AD subscale	19	11	22	9	47.4	0.072
Normal range	50	39	78	10	52.6	

*p*-values calculated using chi-sq or Fisher’s exact test.

## Data Availability

The data for this study are available by contacting the corresponding author.
